# Celiac Disease in Juvenile Idiopathic Arthritis and Other Pediatric Rheumatic Disorders

**DOI:** 10.3390/jcm11041089

**Published:** 2022-02-18

**Authors:** Dimitri Poddighe, Micol Romano, Kuanysh Dossybayeva, Diyora Abdukhakimova, Dinara Galiyeva, Erkan Demirkaya

**Affiliations:** 1 Department of Medicine, Nazarbayev University School of Medicine (NUSOM), Nur-Sultan 010000, Kazakhstan; kuanysh.dossybayeva@nu.edu.kz (K.D.); dabdukhakimova@nu.edu.kz (D.A.); d.galiyeva@nu.edu.kz (D.G.); 2 Clinical Academic Department of Pediatrics, National Research Center for Maternal and Child Health (NRCMCH), University Medical Center (UMC), Nur-Sultan 010000, Kazakhstan; 3 Division of Pediatric Rheumatology, Schulich School of Medicine & Dentistry, University of Western Ontario, London, ON N6A 5W9, Canada; micol.romano@lhsc.on.ca (M.R.); erkan.demirkaya@lhsc.on.ca (E.D.); 4 Department of Epidemiology and Biostatistics, Schulich School of Medicine & Dentistry, University of Western Ontario, London, ON N6A 5W9, Canada

**Keywords:** celiac disease, juvenile idiopathic arthritis, pediatric systemic lupus erythematosus, juvenile dermatomyositis, systemic scleroderma, screening, prevalence

## Abstract

Celiac Disease (CD) is an immune-mediated and gluten-related disorder whose prevalence is higher in children affected with other autoimmune disorders, including diabetes mellitus type 1, autoimmune thyroiditis, and others. As regards Juvenile Idiopathic Arthritis (JIA) and other pediatric rheumatic disorders, there is no clear recommendation for CD serological screening. In this review, we analyze all the available clinical studies investigating CD among children with JIA (and other rheumatic diseases), in order to provide objective data to better understand the necessity of CD serological screening during the follow-up. Based on the present literature review and analysis, >2.5% patients with JIA were diagnosed with CD; however, the CD prevalence in JIA patients may be even higher (>3–3.5%) due to several study limitations that could have underestimated CD diagnosis to a variable extent. Therefore, serological screening for CD in children affected with JIA could be recommended due to the increased CD prevalence in these patients (compared to the general pediatric population), and because these JIA patients diagnosed with CD were mostly asymptomatic. However, further research is needed to establish a cost-effective approach in terms of CD screening frequency and modalities during the follow-up for JIA patients. Conversely, at the moment, there is no evidence supporting a periodical CD screening in children affected with other rheumatic diseases (including pediatric systemic lupus erythematosus, juvenile dermatomyositis, and systemic sclerosis).

## 1. Introduction

Celiac Disease (CD) is an immune-mediated and gluten-related disorder occurring in around 3% of patients who are carriers of specific HLA-DQ alleles (DQA1*0501-DQB1*02 and/or DQA1*0301-DQB1*0302) [1,2]. The hallmark of CD is the development of consistent histopathological alterations of the small bowel mucosa, including increased intraepithelial lymphocytes (IELs), crypts hyperplasia, and shortened/atrophic intestinal villi, as reflected by the Marsh–Oberhuber classification [3]. However, CD is not only a gastrointestinal disorder but also a systemic disease: indeed, the gastrointestinal manifestations are only one component of the clinical picture in these patients (including children), who often display extra-gastrointestinal involvement, including musculoskeletal complaints [4,5,6]. Conversely, there are several reports describing patients affected with rheumatic disorders who resulted to be concomitantly affected with CD and, thus, are claimed to deserve periodical screening for it by some authors [7].

Several autoimmune disorders were included in the European Society for Pediatric Gastroenterology, Hepatology, and Nutrition (ESPGHAN) guidelines for the Diagnosis of Coeliac Disease (published in 2012), as “conditions associated with CD”. Notably, Juvenile Idiopathic Arthritis (JIA) was listed among those in this position paper, where 1.5–2.5% CD prevalence was reported among these patients; however, this information was supported by only two studies dated back to 1996 and 1997, respectively [8,9,10]. Unlike other autoimmune disorders (such as type 1 diabetes mellitus, autoimmune thyroiditis, and autoimmune liver disease), no recommendation was given about CD screening in JIA patients in this position paper; moreover, JIA is not cited at all in the recently updated ESPGHAN guidelines for CD screening and management [8,9,10,11]. However, the growing availability of serological tests for CD promoted and increased its screening in a larger number of children, including those with rheumatic disorders, even though no clear recommendations or guidelines have been published in this regard. This gap of knowledge can cause both CD under-diagnosis (with potentially negative long-term consequences for rheumatic children in whom CD is overlooked) and/or inappropriate use of CD serological tests (with consequent waste of resources and/or diagnostic concerns).

In this review, we analyzed all the available clinical studies investigating CD among children with JIA, in order to provide objective data to better understand the necessity of CD serological screening during the follow-up of this rheumatic disorder. Moreover, in order to make our analysis more complete, we also assessed whether there is any evidence regarding the potential association between CD and other rheumatic diseases in children.

## 2. Juvenile Idiopathic Arthritis and Celiac Disease

Juvenile idiopathic arthritis (JIA) is the most common chronic rheumatic disorder in children. It is diagnosed in patients aged up to 16 years with chronic arthritis (lasting 6 weeks or more), which is not associated to any specific and recognizable etiology (e.g., infectious, neoplastic, metabolic). According to the International League of Associations for Rheumatology (ILAR), five main subtypes can be defined inside the JIA classification: systemic (sJIA); oligoarticular (oJIA), which may be persistent or extended; polyarticular (pJIA), which is usually rheumatoid factor (RF) negative and, much less frequently, positive; psoriatic (PsJIA); and enthesitis-related (ERA). Additionally, JIA may be categorized as undifferentiated, if arthritis does not fulfill the diagnostic criteria for any of the aforementioned subtypes [12]. Like CD, HLA system plays a role in JIA etiopathogenesis; however, the HLA genetic predisposition to JIA is mainly due to other HLA class II molecules (HLA-DRB1, HLA-DPB1), which are differentially involved according to the JIA subtype [13,14].

In order to investigate the epidemiological burden of CD in JIA patients, the literature search was performed using PubMed with the keywords ([“children”] AND “Celiac Disease” AND “arthritis” OR “Juvenile Idiopathic Arthritis”) restricted between 2000 and 2021 (31 December). Only the clinical studies describing cohorts (not case reports or series) of JIA patients screened for CD were included for data extraction, as summarized in Table 1 [15,16,17,18,19,20,21,22,23,24,25,26,27,28].

The study by Stagi et al. reported a relatively high prevalence (6.7%) of CD in their cohort of JIA patients [15]. A comparable result emerged from the research by Skrabl-Baumgartne et al., who found a 4.2% prevalence of CD in their JIA patients [20]. Notably, both studies included a control group, where no CD cases were identified: moreover, these were among the largest studies (JIA: *n* = 151 and *n* = 95, respectively; controls: *n* = 158 and *n* = 100, respectively) of the present selection [15,22]. Actually, in terms of JIA cohort, the second largest research was the prospective study published by Alpegiani et al. (*n* = 108), who reported a CD prevalence of 2.8%; however, no control group was available in this research [16]. A third study (by Stoll et al.) included a control group, but patients’ number (JIA: *n* = 32; controls: *n* = 10) was among the smallest ones; here, no CD diagnoses were made in either group [18].

**Table 1:** Clinical studies assessing CD in children affected with JIA.

Author-1st-(Year) [Country]	Study Design	CD Tests	JIA Pts.	JIA M/F & *Age* *	JIA Duration	CD Pts.	CD Symptoms	Comments
Stagi [15] (2005) [Italy]	CROSS	AGA EmA tTG	151	21/120 *8.3 (2.4–16.9)*	n/a	10 (6.7%)	n/a	CD: M/F = 1/9; oJIA: *n* = 5, pJIA: *n* = 5; Marsh: n/a.
Alpigiani [16] (2008) [Italy]	PRO	EmA tTG	108	37/71 *5.7 (1–15)*	-	3 (2.8%)	No (*n* = 2) Yes (*n* = 1) ^	Annual serological screening Study follow-up: 5.3 yrs. (0.15–12.3) No further information on CD pts.
Koehne [17] (2012) [Brazil]	CROSS	AGA (IgA/IgG) EmA [tTG (IgA)]	32	n/a	n/a	0	-	Only EmA+ patients underwent tTG IgA test.
Stoll [18] (2012) [USA]	CROSS	tTG IgA	42 (*n* = 11) ^E^	11/30 *8.8 (4.6–21) ^E^* *2.8 (1.5–5.9) ^NE^*	2.1 ± 2.2 ^E^ 2.2 ± 2.7 ^NE^	0	-	-
Robazzi [19] (2013) [Brazil]	CROSS	tTG	53	28/25 *10.4 (2.3–17.9)*	3.4 ± 3.1	1 (1.9%)	No	CD: M; 10 yrs; sJIA; Marsh: 3c.
Moghtaderi [20] (2016) [Iran]	CROSS	tTG IgA	53	27/26 *10.6 (1.5–16)*	3.5 ± 3.0	0	-	One patient was anti-tTG IgA+, but endoscopy was normal
Nisihara [21] (2017) [Brazil]	CROSS	EmA tTG IgG	45	16/29 *12 (3–16)*	0.5–10	0	-	Four patients were serologically positive (EmA, *n* = 2; tTG IgG, *n* = 2), but all declined endoscopy.
Skrabl-Baumgartne [22] (2017) [Austria]	CROSS	tTG IgA	95	29/66 *12.3 (2.3–17.9)*	n/a	4 (4.2%)	No (*n* = 3) Yes (*n* = 1) ^	Only tTG IgA+ pts. were tested for EmA (all+) CD: M/F = 2/2; oJIA: *n* = 2, pJIA: *n* = 1, PsJIA: *n* = 1; Marsh 3c: *n* = 2, 3b: *n* = 1, 2b: *n* = 1.
Tronconi [23] (2017) [Italy]	RETRO	tTG IgA	79	28/51 *10.7 (2.8–21)*	-	3 (2.4%)	n/a	annual serological screening CD: M/F = 0:3; oJIA, *n* = 2; PsJIA, *n* = 1.
Sahin [24] (2019) [Turkey]	CROSS	tTG IgA	96	40/56 *11.6 (n/a)*	n/a	0	-	-
Oman [25] (2019) [Sweden]	CROSS	tTG IgA/IgG	216	81/135 *8.4 (3.1–13.3)*	n/a	6 (2.8%)	No (*n* = 3) Yes (*n* = 3) ^	CD: oJIA: *n* = 4, PsJIA, *n* = 1; ERA, *n* = 1; M/F = 0/6; Marsh III: *n* = 6.
AlEnzi [25] (2020) [Saudi Arabia]	CROSS	AGA IgA/IgG EmA tTG	73	28/45 *10.0 ± 2.6*	n/a	1 (1.4%)	n/a	8 pts. had one or more positive CD markers. All underwent endoscopy, but all resulted Marsh neg.
Pagnini [27] (2021) [Italy]	RETRO	tTG/Ema/AGA	53 (ERA)	33/20 *10.9 (3–16)*	-	1 (1.8%)	n/a	Unclear if all patients received serological screening
Sadeghi [28] (2021) [Iran]	CROSS	tTG IgA	78	[1:2] *7.9 (1.6–16)*	2.8 ± 2.8	1 (1.3%)	No	3 pts. resulted tTG IgA+, but only one accepted endoscopy CD: M; oJIA.

* Age is expressed as mean age (age range), except in two articles (Oman et al.: inter-quartile range between brackets; AlEnzi study: mean and standard deviation). ^ Patients already diagnosed with CD before JIA onset; ^E^ JIA patients diagnosed as ERA in the study by Stoll (^NE^ JIA patients diagnosed as non-ERA). Abbreviations: M, male; F, female; pts., patients; AGA, anti-gliadin antibody; EmA, anti-endomysium antibody; tTG, anti-tissue transglutaminase antibody; SpA, Spondylo-arthritis; n/a: information not available; PsJIA: psoriatic arthritis; oJIA: oligoarticular arthritis; pJIA: polyarticular arthritis; sJIA: systemic arthritis; ERA: entesitis-related arthritis; CROSS, cross-sectional study; RETRO, retrospective study; PRO, prospective study.

Among the remaining studies included in our selection, no one included a control group, and CD cases were identified in six studies, showing a CD prevalence ranging between 1.3% and 2.8% [19,23,25,26,27]. All the other studies did not identify any CD patient; however, these were basically the least numerous studies [17,18,20,21], except the one published by Sahin et al. (*n* = 96) [24]. Notably, these studies adopted an incomplete CD screening strategy: indeed, Khoene et al. and Nisihara et al., respectively, performed the serological screening by using anti-gliadin antibody(AGA)/anti-endomysia antibody (EmA) and EmA/anti-tissue transglutaminase IgG (tTG IgG), which are not the most sensitive serological markers for CD [17,21]. Currently, anti-tissue transglutaminase IgA (tTG IgA) is considered the most accurate serological marker (in terms of both sensitivity and specificity) for CD in children [11,29,30]. Sahin et al., Stoll et al., and Moghdateri et al. actually used tTG IgA to screen their JIA patients [18,20,24]; however, some patients may be positive for EmA only (and tTG-IgA negative, at least in the initial stage of disease) [5,31,32] and, therefore, a cross-sectional study where EmA or tTG IgA are not used together may have lost these CD patients, even if they are very few.

Considering the sum of all articles included our literature research, there were 1174 JIA patients, and 30 of them were concomitantly diagnosed with CD, suggesting a CD prevalence as high as 2.6% in JIA patients. This prevalence may be an estimation by defect: in addition to the aforementioned concerns about the serological screening in several studies, some tTG IgA and/or EmA serologically positive patients did not undergo or declined the upper gastrointestinal endoscopy (overall, around 10 serologically positive patients declined this procedure in these studies, whereas this information is not available in at least four studies). Finally, it is worth reporting that the total IgA measurement is clearly confirmed in only half of the studies (7 out of 14), whereas in the remaining ones, such an important aspect is not specified. Indeed, as emphasized by several authors and guidelines, the concomitant measurement of total serum IgA is an essential step for a complete CD serological screening, since IgA deficiency impairs the reliability of the most sensitive markers, namely EmA and tTG IgA [11,33,34]. In this regard, JIA patients could also be characterized by an increased prevalence of IgA deficiency, as it occurs in several autoimmune disorders [33,35]. Unfortunately, recent data investigating IgA levels in JIA are missing, but a dated report by Pelkonen et al. reported >7% frequency of persistent or transient IgA deficiency in their study population (including 350 patients with JIA) [36]. If this finding could be confirmed, that may represent an additional reason for under-estimating CD prevalence in JIA.

In summary, based on the present literature review and analysis, >2.5% patients with JIA were diagnosed with CD; however, considering the suboptimal strategy for CD serological screening and the incomplete diagnostic work-up (without upper GI endoscopy and, thus, duodenal biopsy), as discussed above, the CD prevalence in JIA patients may be even higher (>3–3.5%). Anyway, even the actual CD prevalence of 2.6% emerging from our analysis in JIA patients is higher than CD prevalence in the general pediatric population, which is estimated to be around 1% [37]. Therefore, JIA patients seem to be at greater risk of developing CD during the pediatric age. Even though this increased risk is not as high as in other conditions reported in the aforementioned ESPGHAN CD guidelines (such as type 1 diabetes mellitus, autoimmune thyroiditis, and autoimmune liver disorders) [8], JIA patients could be eligible for periodic CD screening, although the frequency of these serological tests may be debated. Methodologically standardized and controlled prospective studies with larger sample sizes are needed to confirm this number and assess the most cost-effective approach to monitor JIA patients as regards the potential occurrence of CD.

Notably, in the present analysis, all JIA patients concomitantly diagnosed with CD were reported to be asymptomatic for the latter condition (see Table 1), which would further support the indication to screen JIA patients for CD during their rheumatological follow-up. Although this clinical practice has been already implemented in some rheumatological centers, this approach is neither systematic nor standardized since it is not supported by clear evidence. Indeed, as mentioned above, additional research may lead to specific algorithms to screen JIA patients for CD, which could also consider some demographic, clinical, and laboratory parameters to implement a cost-effective approach for this purpose.

As regards the JIA subtypes, among those 30 ascertained CD diagnoses in JIA patients, the JIA subtype is declared for 25 of them (oJIA: *n* = 14, pJIA: *n* = 6; PsJIA: *n* = 3; ERA: *n* = 1; sJIA: *n* = 1). Accordingly, oJIA represents >55% of CD/JIA patients, whereas this subtype is considered to account for around 40% of JIA patients, regardless of the ethnicity; conversely, sJIA is diagnosed in around 13% of JIA patients [38], whereas only 1 JIA patient (4%) was diagnosed with CD. Again, >75% (19 out of these 25) JIA patients diagnosed with CD were female, which corresponds to a female-to-male ratio greater than 3:1, which is higher than the gender ratios previously reported for JIA patients in both European (7:3) and non-European (3:2) populations [38]. Finally, information about additional autoimmune disorders (e.g., thyroiditis) and/or family history and/or some laboratory parameters commonly assessed during JIA follow-up (e.g., liver enzymes, specific autoantibodies, others) might further affect the CD risk in this clinical setting; unfortunately, no data are provided by the aforementioned studies in this regard.

## 3. Pediatric Systemic Lupus Erythematosus and Celiac Disease

Systemic lupus erythematosus (SLE) is an autoimmune disease with very variable expression, since all the organs and systems can be affected [39]. Pediatric SLE (pSLE) is diagnosed in patients younger than 18 years and represents 10–20% of all SLE cases [40]. The immunopathogenesis of SLE is extremely complex and involves both adaptive and innate immune mechanisms [41,42], but a large and heterogeneous production of autoantibodies can be considered a main feature, which is also consistent with its very variable clinical expression and organ/systemic involvement [43,44,45]. Among those, anti-dsDNA antibody is a hallmark of SLE, since it is a very specific and sensitive marker and even correlates with the disease activity [46,47].

Several case reports suggested a possible association between CD and SLE in adults [48,49,50]. An Israelian study based on a large medical database compared 5,018 patients with SLE and 25,090 age- and sex-matched controls and reported an association between SLE and CD with a multivariate OR of 3.92 [51]. Conversely, other studies found no association between SLE and CD [52,53] Very recently, a study by Soltani et al., including both adults and children (without any specific age-related analysis), reported a prevalence of 3% for biopsy-proven CD in patients with SLE and, thus, revamped this issue [54].

In this review, we focused on the pediatric population and, thus, pSLE. Based upon literature research in PubMed database (“children” AND “Celiac Disease” OR “[Juvenile] systemic lupus erythematosus”) starting from 2000 until 2021 (31 December), we could retrieve only a few clinical studies (excluding case reports) assessing the presence of CD in pSLE patients, as summarized in Table 2 [26,55,56].

Unfortunately, these three pediatric studies included small cohorts of patients, and the screening approach was quite variable. Overall, none showed a potential increase in CD prevalence in pSLE patients [26,55,56].

However, a recent study by Shamseya et al., assessed CD serology in a group of 100 SLE adult patients (34.6 ± 9.6 years) who were actually diagnosed during the pediatric age (pSLE at their onset). These (p)SLE patients were compared with a sex- and age-matched control group (*n* = 40). All the study participants were tested for tTG IgA (or tTG IgG, if IgA deficiency was detected); moreover, if anyone was tTG-positive, this patient was tested for EmA IgA too. Briefly, 10 (p)SLE patients (10%) were both tTG and EmA IgA positive and all controls tested negative: such a difference was statistically significant. Upper gastrointestinal endoscopy was performed in all these 10 (p)SLE patients: the histopathological assessment confirmed CD in 6 patients, where 4 cases were labelled as latent CD at that moment [57]. Therefore, even though pSLE does not seem to be associated with CD during the pediatric age, these patients may be at increased risk later in their life.

## 4. Celiac Disease in Other Pediatric Rheumatic Disorders

In the aforementioned study by Aikawa et al., 41 patients with juvenile dermatomyositis (JDM) were included, in addition to those children affected with pSLE. Among them, only one JDM patient was diagnosed as CD [55]. The absence of additional clinical studies assessing CD in JDM patients precludes any conclusion in children. However, O’Callaghan et al. described 51 adult dermatomyositis patients who were tested by AGA IgA, EmA, and tTG IgA: 5 patients were positive for AGA, and 3 of them received histopathological confirmation for CD [58]. Similarly, Orbach et al. showed mildly but significantly higher levels of AGA IgA and anti-tTG IgA levels patients affected with idiopathic inflammatory myopathies (IIMs) compared with matched adult controls, but no histopathological data on duodenal biopsy were provided by these authors [59]. Therefore, further research may be advisable in patients affected with IIMs, including JDM, in order to assess the risk for CD.

In addition to JIA patients, the aforementioned study by Robazzi et al., also investigated 66 children affected with rheumatic fever. One patient was diagnosed with CD [19]. No studies are available in the adult population. Anyway, no plausible biological or medical link between rheumatic fever and CD can be highlighted.

Soylu et al. published a study on CD in children developing Henoch–Schoenlein purpura (IgA vasculitis): among 42 study participants tested by EmA, tTG IgA and anti-deamidated gliadin peptide (DGP) IgA/IgG, seropositivity was detected in 5 children, of whom 2 received histological confirmation for CD (one patient declined the duodenal biopsy) [60]. Further research may be appropriate in this specific clinical setting.

No studies assessed CD in children affected with systemic scleroderma (sSC). However, Nisihara et al. tested 60 adult patients affected with sSC for EmA and AGA IgA/IgG: all samples turned out negative [61]. Similarly, Forbess et al. screened 72 adult SSc patients by testing tTG IgA/IgG and DGP IgA/IgG: three patients tested positive for any markers, but no one was confirmed as being affected with CD [62]. Notably, these studies are in contrast with two previous reports by Luft et al. and Rosato et al., who estimated a CD prevalence of 7% and 8% in their respective cohorts of sSC patients; actually, the first study was not supported by any confirmatory duodenal biopsy [63,64]. Anyway, at the moment, there is no evidence to support any systematic CD screening in children affected with sSC.

## 5. Conclusions

The present literature analysis seems to support the indication of serological screening for CD in children affected with JIA, since the CD prevalence in JIA patients could be around threefold greater than in the pediatric general population, and all those patients diagnosed with CD after JIA onset were mostly asymptomatic. However, evidence-based policies and clear recommendations for CD screening are currently lacking in JIA patients; further research is needed to establish a cost-effective approach in terms of CD screening frequency and strategy over the follow-up for JIA patients. As regards other pediatric rheumatic disorders (including pSLE), at the moment, there is no evidence supporting a periodical CD screening.

## Figures and Tables

**Table 2:** Clinical studies assessing CD in children affected with pSLE.

Author-1st-(Year) [Country]	Study Design	CD Tests	SLE Pts.	SLE M/F & *Age* *	SLE Duration	CD Pts.	CD Symptoms	Comments
Aikawa [55] (2012) [Brazil]	CROSS	EmA	79	33/67 10.3 ± 3.4	4.4 ± 3.7	1	No	Out of 79 pSLE pts. in follow-up, only 41 pts. accepted to participate in this study. CD: F; 12.6 yrs; Marsh: n/a.
Sahin [56] (2019) [Turkey]	CROSS	tTG IgA [EmA]	50	6/44 15.5 ± 3.4	n/a	0	n/a	Only tTG IgA+ patients were tested for EMA. 3 pts. resulted tTG IgA positive but were EmA- and did not undergo endoscopy.
AlEnzi [26] (2020) [Saudi Arabia]	CROSS	AGA (IgA + G) EmA tTG	34	6/28 10.3 ± 2.7	n/a	0	n/a	6 pts. had one or more positive CD markers. All underwent endoscopy, but all resulted Marsh negative.

* Age is expressed as mean and standard deviation. Abbreviations: M, male; F, female; pts., patients; AGA, anti-gliadin antibody; EmA, anti-endomysium antibody; tTG, anti-tissue transglutaminase antibody; CROSS, cross-sectional study.

## Data Availability

Not applicable.
